# The Use of Climatic Niches in Screening Procedures for Introduced Species to Evaluate Risk of Spread: A Case with the American Eastern Grey Squirrel

**DOI:** 10.1371/journal.pone.0066559

**Published:** 2013-07-03

**Authors:** Mirko Di Febbraro, Peter W. W. Lurz, Piero Genovesi, Luigi Maiorano, Marco Girardello, Sandro Bertolino

**Affiliations:** 1 Department of Bioscience and Territory, University of Molise, Pesche, Isernia, Italy; 2 Randersacker, Germany; 3 ISPRA - Institute for Environmental Protection and Research and Chair IUCN Invasive Species Specialist Group, Rome, Italy; 4 Department of Biology and Biotechnologies “Charles Darwin”, University of Rome La Sapienza, Rome, Italy; 5 Centre for Ecology and Hydrology, Wallingford, Oxfordshire, United Kingdom; 6 Department of Agriculture, Forest and Food Sciences, University of Turin, Grugliasco, Turin, Italy; University of Melbourne, Australia

## Abstract

Species introduction represents one of the most serious threats for biodiversity. The realized climatic niche of an invasive species can be used to predict its potential distribution in new areas, providing a basis for screening procedures in the compilation of black and white lists to prevent new introductions. We tested this assertion by modeling the realized climatic niche of the Eastern grey squirrel *Sciurus carolinensis*. Maxent was used to develop three models: one considering only records from the native range (NRM), a second including records from native and invasive range (NIRM), a third calibrated with invasive occurrences and projected in the native range (RCM). Niche conservatism was tested considering both a niche equivalency and a niche similarity test. NRM failed to predict suitable parts of the currently invaded range in Europe, while RCM underestimated the suitability in the native range. NIRM accurately predicted both the native and invasive range. The niche equivalency hypothesis was rejected due to a significant difference between the grey squirrel’s niche in native and invasive ranges. The niche similarity test yielded no significant results. Our analyses support the hypothesis of a shift in the species’ climatic niche in the area of introductions. Species Distribution Models (SDMs) appear to be a useful tool in the compilation of black lists, allowing identifying areas vulnerable to invasions. We advise caution in the use of SDMs based only on the native range of a species for the compilation of white lists for other geographic areas, due to the significant risk of underestimating its potential invasive range.

## Introduction

Species introduction represent one of the main factors in the ongoing biodiversity crisis, with important impacts on ecosystems [1]–[3], and huge economic losses [4], [5]. Given that eradication and control of established populations of introduced species is costly and difficult to implement [6]–[9], strategies aiming at mitigating these impacts should focus on prevention and early warning and rapid response. The implementation of such strategies requires the development and adoption of screening tools designed to identify potentially harmful species before importing them into a country [10], or to facilitate prompt response in the event of new introductions [11]. In particular, the development of black and white lists would help in the first screening of species proposed for import [12], [13]. Such lists should contain species already identified as invasive or that have the potential to be so (black lists), or species classified at low risk following a risk assessment procedure, or based on long-standing experience (white lists). One of the correlates of successful introductions that often emerges, is a match between the ecological conditions of the donor and the invaded areas [14]–[17]. Introduced species have a higher probability of successfully establishing viable populations in areas with a climate that is similar to the native region. According to a precautionary approach, species already established in a country should be banned from other countries with similar ecological and climatic conditions, but for species never introduced before this source of information is lacking. If for instance, we consider the pet trade that is an important vector of introductions for vertebrates [16], [18], it is intuitive that banning species that have already proved to be harmful will stimulate the trade of alternative species, never traded before. An evaluation of these new species that do not have a previous history of introduction is thus required. Furthermore, when recording a new invasion, it is important to have quick screening tools to support decision making in terms of appropriate responses [11].

In recent years, Species Distribution Models (SDMs) have been widely used in many fields, including biodiversity research [19], conservation biology [20] and invasion biology [21], [22]. These models are calibrated on the realized niche [23] and rely on the assumptions that species location data used for modelling are representative of its true climatic requirements; that observed species distributions are in equilibrium with current climate; and that the correct climatic predictors have been included in the model [24]. Many studies have used climatic predictors to model patterns of invasion events [21], [25]. However, the application of SDMs in predicting geography of present and future species invasions requires another fundamental assumption, so-called ‘niche conservatism’. This assumes that the species’ niche maintains its original features over space and time [26]. Assuming niche conservatism, SDMs predict possible invasion events only in areas with similar climatic conditions to the species’ native range. Biological invasions represent an ideal opportunity to verify niche conservatism assumptions, allowing the investigation of spatial niche dynamics in non-native environments, a process usually occurring over time. Thus many recent studies used SDMs to evaluate differences in species’ niches between the native and non-native range [21], [25], [27]–[29].

The overall objective of this study was to provide a foundation for screening procedures that will use the realized climatic niche of a species in its native range in order to evaluate its adaptability in new areas. If the realized climatic niche of a species could be used to predict the areas of the world where it could adapt, then this modelling procedure would be an economic and effective tool in compiling black and white lists to prevent new introductions.

We test this assertion using the American eastern grey squirrel (*Sciurus carolinensis*) (henceforth simply referred to as ‘grey squirrel’), a rodent of the family Sciuridae that has been successfully introduced into many countries, as a case study. The grey squirrel naturally occurs in the Eastern side of North America, ranging from the Mexican Gulf to Southern Canada [30]. It has been introduced to many localities of North America, Australia (now extinct), South Africa, Great Britain, Ireland and Italy [18], [31], [32]. At present, the range of the grey squirrel in Europe covers most of England and Wales, part of Scotland, the Eastern part of Ireland, as well as extensive areas in North Western and Central Italy [33]–[35].

The grey squirrel represents a serious threat for biodiversity in its non-native range. Its spread in the British Isles and in Northern Italy is causing the progressive decline of the native red squirrel (*Sciurus vulgaris*) through disease mediated competition [36]–[38]. It also causes significant and costly damage to forests and tree plantations, and may prey on eggs and chicks of many forest birds [39], [40]. A modelling approach has been used to predict the grey squirrel population expansion at a regional and local scale, but this approach has relied on limited life history information for the species in its new range and did not consider climatic factors [41]–[44]. We ask the following questions: i) Does the grey squirrel introduced into Europe maintain the same realized climatic niche as in its native range? ii) What are the areas at risk of invasion at a global level? iii) How useful can SDMs be in compiling black and white lists and estimating potential distribution of invasive alien species?

## Materials and Methods

### Species Records

**Figure 1:**
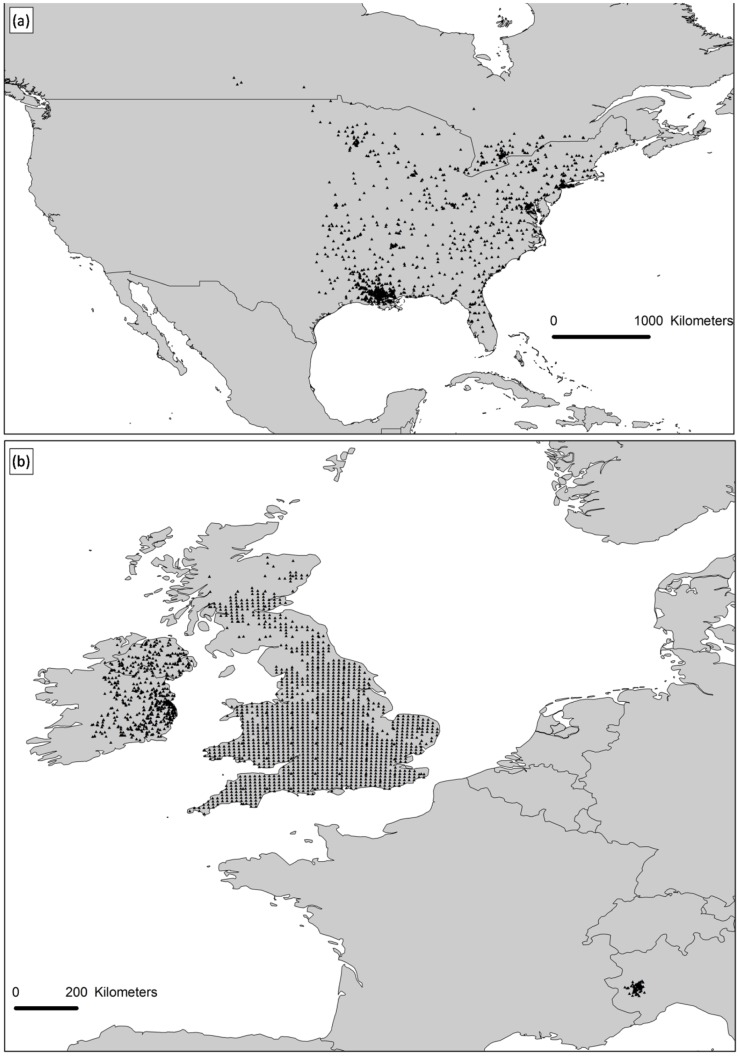
Occurrences of *S. carolinensis* in native range (a) and invasive range (b), used to calibrate models.

Species occurrences for both invaded and native ranges were derived from different resources, including online databases as the Global Biodiversity Information Facility (GBIF, http://www.gbif.org), the Mammal Networked Information System (MaNIS, http://manisnet.org/) and the specimen online collections of “American Museum of Natural History” (http://www.amnh.org/), “Smithsonian Institution National Museum of Natural History” (http://www.mnh.si.edu/) and “Royal Ontario Museum” (http://www.rom.on.ca/). Species records for Piedmont (Northern Italy) were provided by S.B. (unpublished data). Occurrences without coordinates were georeferenced using BioGeomancer [45], if a geographic indication equivalent to an administrative level of “municipality” (as intended by the Darwin Core data standardization system; http://terms.gbif.org/wiki/dwc:municipality) was provided, otherwise they were discarded. Duplicate records falling into the same municipality boundaries were excluded. Records not falling into the IUCN native range [46] were excluded from the dataset. For the invasive range, records from United Kingdom, Ireland and Northern Italy were used. These countries represent the oldest areas of introduction, with documented self-sustaining populations, which colonized both natural and man-modified habitats. We collected a total of 2997 records of presence, 981 for the native range and 2016 for the invasive one (Fig. 1).

### Climate Data

The selection of environmental predictor variables in SDMs is often a function of the scale of the analysis; but in general, the predictors describing the physical environment often fall into three classes: 1) climate, 2) topography, and/or 3) land use. The predictive power of SDMs at broad scales may not be substantially improved by including variables other than climate [47], but land-use and topographic variables related to direct and resource gradients, may be more important at finer, regional scales [48], [49]. Although it is undeniable that non-climatic factors related to the physical environment and interspecific dynamics (i.e. competition, predation) play major a role in determining the distribution of the grey squirrel, an analysis based only climatic variables can give a first good approximation of distribution of a species at the global scale [49].

Climate data were obtained from the Worldclim database [50]. Worldclim contains interpolated surfaces for 19 climatic variables, available at different spatial resolutions. Given that our aim was to broadly screen for climatically suitable areas for the grey squirrel, across the main geographic regions of the world, we decided not to use the finest resolution data available within Worldclim (30 arc-second resolution). We decided instead to use the dataset with a 2.5 arc-minutes resolution. We chose a subset of the climatic variables on the basis of what is known about the grey squirrel ecology and our knowledge of the species. The grey squirrel seems to be primarily limited by low temperatures and abundant precipitations, especially during winter. The severity of winter weather was negatively associated with population size of grey squirrels and survival from summer to winter [51]. A severe frost could reduce food availability and food quality determining lighter body weight, reduced reproductive rate and increased mortality in young and subadults [52]. [51] confirmed the interaction between food availability and winter climate, showing that the severity of winter weather could mask the positive effects of tree seed availability limiting grey squirrel densities. These effects are indirectly confirmed by the limited spread of the grey squirrel introduced to West Scotland, a region characterized by heavy rains and low temperatures [33], [53]; see also Fig. 1. We thus selected all the variables related to winter temperature and precipitations. Because of potential problems with multi-collinearity [54] we further reduced our variables to four: Mean Temperature of the Wettest Quarter (MTWQ); Mean Temperature of the Coldest Quarter (MTCQ); Precipitation of the Wettest Quarter (PWQ); Precipitation of the Coldest Quarter (PCQ). These variables were retained as they showed a correlation of 0.80 or less as measured by the Pearson’s correlation coefficient. Pearson’s correlation tests were performed using statistical software R 2.14.2 [55].

### Modelling Approach

All the models were calibrated using Maxent [56], [57]. Maxent is a machine - learning method that estimates species distributions using environmental predictors together with species occurrences. This algorithm, based on an application of the maximum entropy principle in an ecological context [58], estimates the distribution probability in such a way as to satisfy a set of constraints derived from environmental conditions at species’ presence sites. These constraints impose that the expected value of each environmental predictor falls as close as possible to the empirical mean of that predictor measured over the presence records. Between all the possible distributions that satisfy constraints, the algorithm chooses the closest to the uniform, maximizing the entropy. Maxent has generally shown to perform better than other similar techniques, especially in predicting invasive species distributions outside their native ranges [59]–[61].

We kept the default settings in the algorithm, with the exception of the number of replicates and default prevalence. In order to obtain a reliable evaluation of the model, we randomly split the occurrence data into two subsets, using 70% of records to calibrate the model and the remaining 30% to evaluate the model. This procedure was replicated 10 times, each time randomly selecting different 70% - 30% portions of occurrence data. The final model was obtained averaging the 10 runs. For each replicate, we evaluated the predictive performance of the models by calculating the area under curve (AUC), the sensitivity, specificity and true skill statistic (TSS). All metrics were calculated using the R package “PresenceAbsence” [62].

Moreover, to account for the fact that the species is quite common and easy to observe across most of its geographical range, we set the default prevalence to 0.7.

### Native, Invasive and Reciprocal Models

We calibrated three different models: one considering only records from the native range (NRM), the second including records from native and invasive range (NIRM), the third, so-called “reciprocal” [21], [25], calibrated with invasive occurrences and projected in the native range (RCM). For the computation of the NRM, background points were randomly placed in the entire North American continent, assuming this area to be potentially available for the species in terms of dispersal but not entirely occupied because of climatic constraints. Background points for the NIRM were randomly placed also in United Kingdom, Ireland and Piedmont (Northern Italy). Because the majority of the records available to us were derived from the invasive range (2016 against 981), they were not used as a whole to calibrate NIRM. In order to decrease the potential bias occurring from invasive range being over-represented [21], 10 subsets of 10% of the records from the invasive range were randomly selected and a single NIRM has been calibrated from each selection, then the results and the performance metrics were averaged. The three models were then projected worldwide.

The main vector of squirrel’s introductions is the pet trade and the subsequent release of captive animals [18]. Therefore, countries at risk of invasion are those where the grey squirrel is imported. A complete list of countries where squirrels are traded is not available and theoretically grey squirrels could be traded everywhere. For this reason, our predictions considered all countries and not only those where grey squirrels were already introduced or where their trade is reported.

### Niche Overlap Among Native and Invasive Range

Comparisons of the climatic niches, between the native and invasive range, were carried out using the analytical framework proposed by [63]. The analysis of two environmental niches follows three steps:

i) Data preprocessing. In this step the environmental space, as obtained from a multivariate ordination using the climatic data or from the prediction of a species distribution model is divided in cells, each representing a unique vector of environmental conditions ν_ij_, occurring at one or more sites in the geographical space. A Kernel density function [64], is then employed to calculate the density of number of occurrences and number of sites with particular environmental conditions ν_ij_, for each cell of the environmental space. These densities are divided respectively by the maximum number of occurrences in any one cell of the environmental space, max(n_ij_), and by the number of sites with the most common environment, max(N_ij_), as follows:
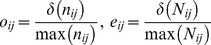
(1)
*o*_ij_ and *e*_ij_ represent two indexes which range from 0, for environments without occurrences nor sites, to 1, for environments with maximum number of occurrences and sites. Then *z*_ij_, the occupancy of each environment ν_ij_ by the species, is calculated as follows:



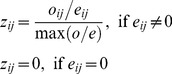
(2)This index also ranges from 0 to 1 and allows unbiased comparisons of occurrence densities between different entities occurring in ranges with environments not equally available [63].

This data preprocessing step overcomes problems related to the spatial resolution of the data, corrects observed occurrence densities for each region (invasive vs. native) in light of the availability of environmental space and most importantly, makes optimal use of both geographical and environmental spaces.

ii) Calculation of the niche overlap measure. In this step the niche overlap in the environmental space is measured, using a revised version of the *D* metric [65], as follows:
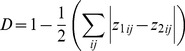
(3)


*D* metric provides a calculation of the niche overlap between two species by comparing their occupancy values, *z*_ij_, calculated in equation 2, over the environmental space.

iii) Performing tests of niche equivalency and similarity [66]. Both equivalency and similarity tests are based on a randomization process. Niche equivalency test evaluates if the niches of two species are effectively identical or if niche overlap value measured between them could be simply due to chance. This test requires the calculation of a null distribution of 100 simulated niche overlap values, obtained by pooling the occurrences of the two species, randomly splitting them with the same proportions of the original dataset and quantifying niche overlap values between each couple of simulated niches. If the observed niche overlap value falls outside the 95% of the null distribution of simulated values, equivalency of the two niches can be rejected. In a similar manner, niche similarity test evaluates how much similarities/dissimilarities between niches of two species can be ascribed to ecological issues (habitat selection and/or suitability) or simply to chance. This second test also involves the calculation of a null distribution of 100 simulated niche overlap values, whereas, in this case, it is obtained shifting randomly the center of the observed distribution of occurrences in one species’ range and calculating niche overlap values between the simulated niches and the observed niche calculated in the other species’ range (range 1 → range 2). The procedure is also carried out in the opposite direction, simulating niches in range 2 and calculating niche overlap with observed niche in range 1 (range 1 ← range 2). If the observed niche overlap value is greater/smaller than the null distribution of the simulated values, the two niches are more similar/different than expected by chance [63], [66].

Broennimann’s framework makes use of a number of ordination and SDMs methods to compare environmental niches (see above mentioned step i). Here we decided to focus on ordination methods considered more appropriate than SDMs to investigate niche overlap [63]. We applied all the ordination methods suggested by [63]. However, as they all yielded similar results only those obtained from the Principal Component Analysis calibrated on the entire environmental space of the two ranges including species occurrences (PCA - env) were reported. Other methods and results are provided in Supplementary Table S1. Following [63] the PCA’s were calibrated both using data from the native range and then projecting results into the invasive range, and using data from both ranges as a whole.

## Results

### Maxent Modelling

Both NRM and NIRM showed excellent predictive abilities (AUC >0.9) according to [67]. The predictive ability of the RCM was poor (AUC <0.7). The inclusion of occurrences from the invasive range of the species had little impact on the predictive power of the model, with the AUC changing from 0.922 for NRM to 0.910 for NIRM. Threshold - dependent measures also showed the NRM as the best model in terms of predictive performance, with 94.3% of presences and 86.1% of absences correctly predicted, and a TSS value of 0.80, consistently better than random [68] (Table 1).

**Table 1:** Results of model validation.

Model	Area of calibration	AUC	Sensitivity	Specificity	TSS
NRM	North America	0.922	0.943	0.861	0.805
RCM	UK, Ireland, Piedmont (Italy)	0.652	0.746	0.514	0.261
avNIRM	North America+UK, Ireland, Piedmont (Italy)	0.910* (sd = 0.0008)	0.942*	0.860*	0.802*

NRM = Native Range Model, RCM = Reciprocal Model, avNIRM = averaged Native+Invasive Range Model.

“*”indicates averaged values.

**Figure 2:**
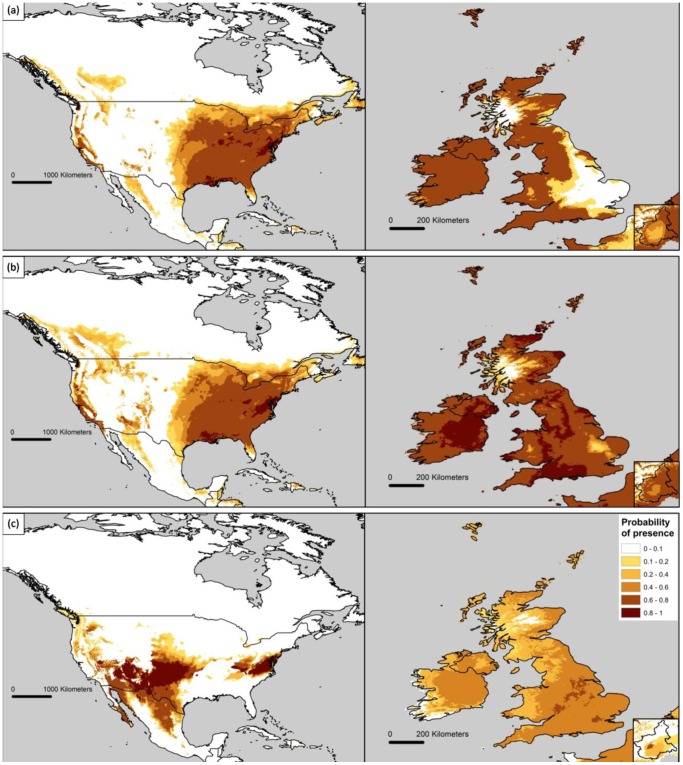
Predicted distribution of the grey squirrel for North America, United Kingdom, Ireland and Piedmont, as obtained from the Native Range (a), Native+Invasive Range (b), and Reciprocal Models (c).

**Figure 3:**
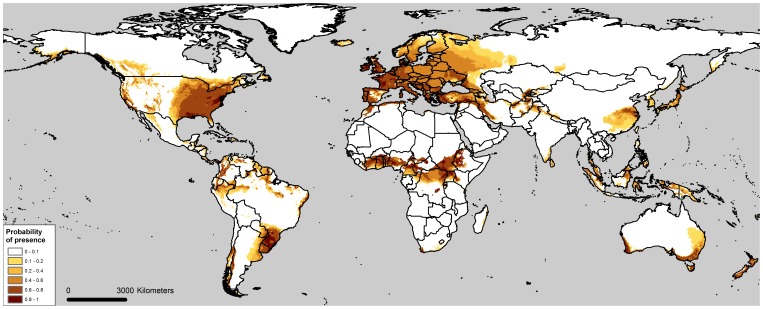
Worldwide projections of grey squirrel’s climatic niche calculated by NIRM.

The geographical projections of the grey squirrel’s climatic niche predicted by the three models showed that NRM failed to predict a large extent of the invasive range in the Eastern parts of the United Kingdom, whereas it accurately predicted an unsuitable area in Western Scotland, where the species is absent. The RCM highly underestimated and shifted the native range of the species westwards in North America. NIRM accurately predicted both the native and invasive range, including the lack of suitable climatic conditions in Western Scotland (Fig. 2). Worldwide projections of the NIRM predicted many highly suitable areas on all continents, including a large extent of Europe and Subsaharan Africa, areas of South Eastern Asia and South America and South Eastern regions of Australia, identifying these as highly vulnerable areas in case of grey squirrel’s introduction (Fig. 3).

### Niche Shift

Within the ordination techniques used to calculate niche overlap (see Table S1 in Supporting Information), PCA - env showed the highest niche overlap value, both when calibrated using data from the native range and when coupling these data with occurrences from invasive range. Niche equivalency hypothesis was rejected in both cases, revealing significant differences between the grey squirrel’s niche in native and invasive ranges. Niche similarity yielded no significant results, leading to nonrejection of the null hypotheses of niche similarities due to chance (Table 2).

**Table 2:** Results of niche shift analysis.

Technique	Area of calibration	*D* metric	Niche equivalency test significance level	Niche similarity test (range 1→range 2) significance level	Niche similarity test (range 1←range 2) significance level
PCA - env	Native+Invasive range	0.208	<0.01	ns	ns
PCA - env*	Native range	0.243	<0.01	ns	ns

“*” indicates methods calibrated in native range and projected in invasive range. *D* metric quantifies niche overlap (Schoener, 1970). Arrows specify directions of niche similarity test (*see text*).

**Figure 4:**
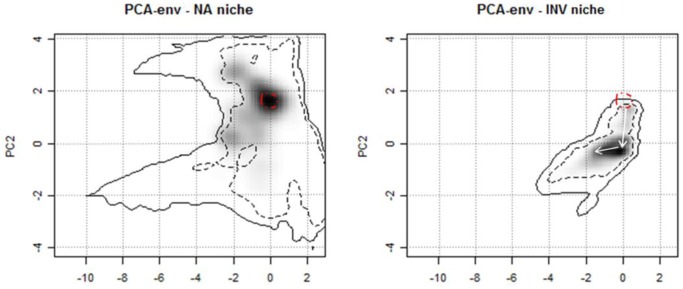
The grey squirrel’s niche in climatic space, calculated with the PCA - env method. The surfaces in the plots represent the climatic niche along the first two axis of the PCA in native (left) and invasive (right) range. Grey shading indicates the density of species occurrences. Solid and dashed lines represent, respectively, 100% and 50% of the available environment. Dashed circle indicates the niche center and arrows show its movement in climatic space between native and invasive range.

Results of PCA - env, defined as the best method [63] emphasise how the grey squirrel’s niche center underwent a shift in its position moving toward colder environments when colonizing the non-native range, moreover expanding its shape with an inclusion of wetter environments (Fig. 4).

## Discussion

Species Distribution Models estimate climatic requirements of a species building its realized niche and could be used to predict the potential risk and extent of spread of species introduced into new areas [61], [69]. The ability to perform an effective screening of species is based on the assumption of ‘niche conservatism’, that a species maintains its native niche over space and time [26]. We tested this hypothesis with the grey squirrel investigating the overlap between native and invasive climatic niches.

### Model Performance

The analyses performed in this study showed that invasive populations of the grey squirrel in Europe occupy climatic conditions not represented in the native distribution of the species. They occurred in colder and wetter areas compared to their native range. This supports the hypothesis of a shift in the species’s climatic niche in the area of introductions. The result was confirmed by the failure of the model calibrated with native occurrences (NRM) and the one calibrated with invasive occurrences (RCM) in predicting respectively invasive and native distributions and by the detection of a statistically significant difference between native and invasive climatic niche.

Although both NRM and the model calibrated pooling native and invasive occurrences (NIRM) offered excellent AUCs, they showed very different performances in predicting native and invasive distributions. NRM accurately predicted the native distribution in North America, whereas it partially failed to predict the invasive distribution in UK and Piedmont. The NRM in particular, predicted the species to be absent from Western Scotland, a cold and rainy area which has actually remained unoccupied by grey squirrel, despite the fact that this region was among the first introduction sites in the UK (introduction at Loch Long, Western Scotland in 1892 [53]). On the other hand, NRM failed to predict the distribution of grey squirrels in South Eastern England, where the species has successfully spread during the last century [34]. In addition, it predicted a large unsuitable area in Piedmont, where the grey squirrel is currently still expanding its range [44]. The NIRM method performed consistently better than the NRM, accurately predicting both native and invasive distributions. Predictions calculated by NIRM correctly described the distribution of grey squirrels in UK and Piedmont, and accurately predicted the species’ absence in Western Scotland. Moreover, NIRM correctly predicted invasive distribution of the species in South Africa, where the species has been confined to the Western Cape region and unable to colonize natural habitats and expand its range [70], [71]. Grey squirrels introduced to Australia went extinct (Melbourne and Ballarat, Victoria) or were eradicated (Adelaide, South Australia) [32]; NIRM predicted unsuitable areas near Melbourne and Adelaide, whereas Ballarat was incorrectly predicted as suitable. Based on these predictions, large areas of Western Europe, Central Africa, Brazil and South Australia are considered highly suitable for the grey squirrel, highlighting the need to develop regional and national invasive species strategies that restrict the importation and trade of this species. Considering that warmer and drier conditions seem to favor the spread of the grey squirrel, the present climate change may further benefit the species in new areas of introductions.

The greater reliability shown by NIRM compared to NRM confirmed results reported in other studies [27], [72], where it is emphasised that SDMs calibrated with occurrences both from native and invasive ranges are more accurate in identifying areas vulnerable to future introductions. [69] argued that taking into account both ranges allows the fitted realized niche to better approximate the fundamental niche of the species. The failure of NRM and RCM in predicting respectively invasive and native distributions supports findings by other studies [21], [25], [27], [29] where the failure was ascribed to a shift in species’ niche during the invasion process. However, statistical methods implemented in the previous studies to fit niches and test significance of potential shifts (SDMs and Principal Component Analysis in [25]; SDMs, PCA and Between - Class Analysis in [27] and [21]; SDMs, PCA and niche equivalency and niche similarity tests *sensu* [66] in [29]) have been criticized of being affected by the availability and distribution of environmental gradients in native and invasive ranges, as all these techniques start from the recorded occurrences of species [63], [73].

### Niche Conservatism

Our study represents one of the first applications of the framework implemented by [60] which overcomes the previous limitations, providing an unbiased evaluation of the dynamics of grey squirrel’s climatic niche during the invasion process. In fact, a strong non-equivalency between native and invasive niches, confirmed by 7 ordination methods and 3 SDMs methods (see Supplementary Table S1), supports the hypothesis of a shift in the species’ climatic niche toward colder and wetter environments, illustrating the potential and ability of species to adapt to new environments. The analyses carried out in this study do not allow the attribution of the resulted niche shift to a realized versus a fundamental niche without experiments on physiological limits of the species. It is therefore not possible to discriminate if native grey squirrel populations are already preadapted to colder and wetter climates, although they do not occupy them in their native range due perhaps to dispersal limitations or competitors/predators pressure, or if micro-evolutionary changes and adaptions to the new environments occurred during and post the invasion process [72]. [27] argued that *Centaurea maculosa* shifted its climatic niche directly in the invasive range. The SDM calibrated with invasive occurrences and projected in native range did not predict any highly suitable areas within the observed range of the species, leading authors to exclude the existence of subpopulations preadapted to the novel climatic conditions of the invasive range.

The RCM calibrated in this study showed the opposite result, predicting a large suitable area within the grey squirrel’s native range (Ohio, Alabama, Maryland, New Jersey, Virginia, West Virginia, North Carolina, South Carolina, Tennessee, Kentucky) and suggesting the existence of subpopulations preadapted to colder and wetter climates. However, the poor predictive performance of RCM (AUC <0.7) did not allow us to make predictions with a high level of confidence with respect to this hypothesis. As argued by other authors [74], [75] high levels of intraspecific genetic variability could make species able to adapt quickly to environmental changes (or new environments in the case of introduced species). The existence of native subpopulations preadapted to wetter and colder climates, coupled with the release from a community in North America composed of many squirrel species [76] could have limited the distribution of the grey squirrel in its native range and allowed introduced individuals to spread in novel climates. The latter would suggest a shift in the realized niche. Multiple introduction events in UK [77] could also have provided high levels of intraspecific genetic variability, facilitating the adaptation of the grey squirrel to habitats in the British Isles.

### Conclusions

In agreement with similar published studies [27], [29], NRM accurately predicted areas of successful introductions in UK, Ireland, Piedmont, South Africa and Australia, but failed to predict patterns of subsequent species spread. The results confirmed how SDMs could be a useful tool for identifying areas vulnerable to invasions. However the greater reliability of NIRM and a statistically significant niche shift between grey squirrel’s native and invasive range also suggest some limitations of SDMs when applied in risk assessments for invasive alien species. We clearly showed that an introduced mammal species can colonize different environments compared to those occurring in its native range. Our work is based only on a single species, and further research is needed before being able to generalize the results obtained. However, based on our findings we recommend caution in using SDMs for the compilation of white lists of species [12]. Particularly, risk assessment based only on the native range climatic niche is likely to underestimate suitable areas and the ability of a species to change its climatic niche. However, SDMs accurately predicted areas for successful introduction events and could be a useful tool to compile so-called black lists, that are lists which identify high risk alien species. They also represents a useful tool especially for a preliminary screening of the invasion risk or to guide decision making in case of early detection of new introductions.

## Supporting Information

Table S1 **Results of niche shift analysis performed using all ordination and SDMs methods proposed by Broennimann et al., (2011).** “*” indicates methods calibrated in native range and projected in invasive range. *D* metric quantifies niche overlap (Schoener, 1970). Arrows specify if a niche similarity test was performed, simulating niches in range 1 and calculating niche overlap with observed niche in range 2, or vice versa.
(DOCX)

## References

[1] Olden JD, LeRoy Poff N, Douglas MR, Douglas ME, Fausch KD (2004) Ecological and evolutionary consequences of biotic homogenization. Trends in Ecology and Evolution Evolution 19: 18–24. Available: http://www.sciencedirect.com/science/article/pii/S016953470300288X. Accessed 18 May 2013. 10.1016/j.tree.2003.09.010 16701221

[2] Clavero M, García-Berthou E (2005) Invasive species are a leading cause of animal extinctions. Trends in Ecology and Evolution 20: 110. Available: http://www.ncbi.nlm.nih.gov/pubmed/16701353. Accessed 2 March 2012. 10.1016/j.tree.2005.01.003 16701353

[3] Vilà M, Basnou C, Pyšek P, Josefsson M, Genovesi P, et al. (2009) How well do we understand the impacts of alien species on ecosystem services? A pan-European, cross-taxa assessment. Frontiers in Ecology and the Environment 8: 135–144. Available: http://dx.doi.org/10.1890/080083. Accessed 18 May 2013.

[4] Kettunen M, Genovesi P, Gollasch S, Pagad S (2008) Technical support to EU strategy on invasive alien species (IAS)-Assessment of the impacts of IAS in Europe and the EU (final module report for the European Commission). Institute for European Environmental Policy (IEEP), Brussels, Belgium. 44 pp.+Annex.

[5] Pimentel D, Zuniga R, Morrison D (2005) Update on the environmental and economic costs associated with alien-invasive species in the United States. Ecological Economics 52: 273–288. Available: http://www.sciencedirect.com/science/article/pii/S0921800904003027. Accessed 18 May 2013.

[6] Bomford M, O’Brien P (1995) Eradication or control for vertebrate pests? Wildlife Society Bulletin 23: 249–255. Available: http://www.jstor.org/stable/10.2307/3782799. Accessed 6 July 2012.

[7] Genovesi P (2007) Limits and Potentialities of Eradication as a Tool for Addressing Biological Invasions. In: Nentwig W, editor. Biological invasions. Ecological studies, Vol. 193. Springer Berlin Heidelberg, Vol. 193: 385–402.

[8] Genovesi P (2011) Are we turning the tide? Eradications in times of crisis: how the global community is responding to biological invasions. In: Veitch CR, Clout MN, Towns DR, editors. Island invasives: eradication and management. IUCN, Gland, Switzerland. 5–8. Available: http://www.issg.org/pdf/publications/Island_Invasives/pdfwebview/0cGenovesiKeynote.pdf. Accessed 6 July 2012.

[9] Panzacchi M, Cocchi R, Genovesi P, Bertolino S (2007) Population control of coypu Myocastor coypus in Italy compared to eradication in UK: a cost-benefit analysis. Wildlife Biology 13: 159–171.

[10] Keller RP, Lodge DM, Finnoff DC (2007) Risk assessment for invasive species produces net bioeconomic benefits. Proceedings of the National Academy of Sciences 104: 203–207. Available: http://www.pnas.org/content/104/1/203.abstract. Accessed 18 May 2013. 10.1073/pnas.0605787104 PMC1765435 17190819

[11] Genovesi P, Scalera R, Brunel S, Roy D (2010) Towards an early warning and information system for invasive alien species (IAS) threatening biodiversity in Europe. Copenaghen, Denmark: European Environmental Agency. Available: http://scholar.google.com/scholar?hl=en&btnG=Search&q=intitle:Towardsanearlywarningandinformationsystemforinvasivealienspecies(IAS)threateningbiodiversityinEurope#0. Accessed 6 July 2012.

[12] Genovesi P, Shine C (2004) European Strategy on Invasive Alien Species: Convention on the Conservation of European Wildlife and Habitats (Bern Convention). Nature and Environment, Vol 137. Bern, Switzerland: Council of Europe Publishing. Available: https://69.90.183.227/doc/external/cop-09/bern-01-en.pdf. Accessed 18 May 2013.

[13] Essl F, Nehring S, Klingenstein F, Milasowszky N, Nowack C, et al. (2011) Review of risk assessment systems of IAS in Europe and introducing the German–Austrian Black List Information System (GABLIS). Journal for Nature Conservation 19: 339–350. Available: http://www.sciencedirect.com/science/article/pii/S1617138111000513. Accessed 18 May 2013.

[14] Jeschke JM, Strayer DL (2006) Determinants of vertebrate invasion success in Europe and North America. Global Change Biology 12: 1608–1619. Available: http://dx.doi.org/10.1111/j.1365-2486.2006.01213.x. Accessed 18 May 2013.

[15] Hayes K, Barry S (2008) Are there any consistent predictors of invasion success? Biological Invasions 10: 483–506. Available: http://dx.doi.org/10.1007/s10530-007-9146-5. Accessed 18 May 2013.

[16] Bomford M, Darbyshire RO, Randall L (2009) Determinants of establishment success for introduced exotic mammals. Wildlife Research 36: 192–202. Available: http://dx.doi.org/10.1071/WR08055. Accessed 18 May 2013.

[17] Van Wilgen NJ, Richardson DM (2012) The Roles of Climate, Phylogenetic Relatedness, Introduction Effort, and Reproductive Traits in the Establishment of Non-Native Reptiles and Amphibians. Conservation Biology 26: 267–277. Available: http://dx.doi.org/10.1111/j.1523-1739.2011.01804.x. Accessed 18 May 2013. 10.1111/j.1523-1739.2011.01804.x 22236256

[18] Bertolino S (2009) Animal trade and non-indigenous species introduction: the world-wide spread of squirrels. Diversity and Distributions 15: 701–708. Available: http://dx.doi.org/10.1111/j.1472-4642.2009.00574.x. Accessed 18 May 2013.

[19] Thuiller W, Lavorel S, Sykes MT, Araújo MB (2006) Using niche-based modelling to assess the impact of climate change on tree functional diversity in Europe. Diversity and Distributions 12: 49–60. Available: http://doi.wiley.com/10.1111/j.1366-9516.2006.00216.x. Accessed 13 March 2012.

[20] Maiorano L, Falcucci A, Zimmermann NE, Psomas A, Pottier J, et al. (2011) The future of terrestrial mammals in the Mediterranean basin under climate change. Philosophical transactions of the Royal Society of London Series B 366: 2681–2692. Available: http://www.pubmedcentral.nih.gov/articlerender.fcgi?artid=3140741&tool=pmcentrez&rendertype=abstract. Accessed 29 March 2012. 10.1098/rstb.2011.0121 PMC3140741 21844047

[21] Beaumont LJ, Gallagher R V, Thuiller W, Downey PO, Leishman MR, et al. (2009) Different climatic envelopes among invasive populations may lead to underestimations of current and future biological invasions. Diversity and Distributions 15: 409–420. Available: http://doi.wiley.com/10.1111/j.1472-4642.2008.00547.x. Accessed 13 March 2012.

[22] Ficetola GF, Thuiller W, Padoa-Schioppa E (2009) From introduction to the establishment of alien species: bioclimatic differences between presence and reproduction localities in the slider turtle. Diversity and Distributions 15: 108–116. Available: http://dx.doi.org/10.1111/j.1472-4642.2008.00516.x. Accessed 18 May 2013.

[23] Hutchinson GE (1957) Concluding remarks. Cold Spring Harbor Symposium on Quantitative Biology 22: 415–427 doi:10.1007/s00726-011-1022-z

[24] Guisan A, Zimmermann NE (2000) Predictive habitat distribution models in ecology. Ecological Modelling 135: 147–186. Available: http://linkinghub.elsevier.com/retrieve/pii/S0304380000003549. Accessed 18 May 2013.

[25] Fitzpatrick MC, Weltzin JF, Sanders NJ, Dunn RR (2007) The biogeography of prediction error: why does the introduced range of the fire ant overpredict its native range? Global Ecology and Biogeography 16: 24–33. Available: http://onlinelibrary.wiley.com/doi/10.1111/j.1466-8238.2006.00258.x/full. Accessed 19 June 2012.

[26] Peterson AT (2003) Predicting the geography of species’ invasions via ecological niche modeling. The Quarterly review of biology 78: 419–433. Available: http://www.ncbi.nlm.nih.gov/pubmed/14737826. Accessed 18 May 2013. 10.1086/378926 14737826

[27] Broennimann O, Treier U, Müller-Schärer H, Thuiller W, Peterson AT, et al. (2007) Evidence of climatic niche shift during biological invasion. Ecology Letters 10: 701–709. Available: http://www.ncbi.nlm.nih.gov/pubmed/17594425. Accessed 18 May 2013. 10.1111/j.1461-0248.2007.01060.x 17594425

[28] Rödder D, Lötters S (2009) Niche shift versus niche conservatism? Climatic characteristics of the native and invasive ranges of the Mediterranean house gecko (Hemidactylus turcicus). Global Ecology and Biogeography 18: 674–687. Available: http://doi.wiley.com/10.1111/j.1466-8238.2009.00477.x. Accessed 13 March 2012.

[29] Medley KA (2010) Niche shifts during the global invasion of the Asian tiger mosquito, Aedes albopictus Skuse (Culicidae), revealed by reciprocal distribution models. Global Ecology and Biogeography 19: 122–133. Available: http://doi.wiley.com/10.1111/j.1466-8238.2009.00497.x. Accessed 7 March 2012.

[30] Koprowski JL (1994) Sciurus carolinensis. Mammalian Species 480: 1–9.

[31] Gurnell J, Pepper H (1993) A critical look at conserving the British Red Squirrel Sciurus vulgaris. Mammal Review 23: 127–137. Available: http://dx.doi.org/10.1111/j.1365-2907.1993.tb00424.x. Accessed 18 May 2013.

[32] Peacock DE (2009) The grey squirrel Sciurus carolinensis in Adelaide, South Australia: its introduction and eradication. The Victorian Naturalist 126: 150–155. Available: http://search.informit.com.au/documentSummarydn=656054952834119res=IELHSS. Accessed 7 July 2012.

[33] Bertolino S (2008) Introduction of the American grey squirrel (Sciurus carolinensis) in Europe: a case study in biological invasion. Current Science 95: 903–906.

[34] Gurnell J, Kenward RE (2008) Grey squirrel Sciurus carolinensis. In: Harris S, Yalden DW, editors. Mammals of the British Isles: Handbook 4th Edit. Southampton, England: The Mammal Society. 66–72.

[35] Martinoli A, Bertolino S, Preatoni D, Balduzzi A, Marsan A, et al. (2011) Headcount 2010: the multiplication of the grey squirrel populations introduced to Italy. Hystrix, the Italian Journal of Mammalogy 21: 127–136. Available: http://www.italian-journal-of-mammalogy.it/article/view/4463. Accessed 7 July 2012.

[36] Sainsbury AW, Nettleton P, Gilray J, Gurnell J (2000) Grey squirrels have high seroprevalence to a parapoxvirus associated with deaths in red squirrels. Animal Conservation 3: 229–233. Available: http://dx.doi.org/10.1111/j.1469-1795.2000.tb00107.x. Accessed 18 May 2013.

[37] Gurnell J, Wauters LA, Lurz PWW (2004) Alien species and interspecific competition: effects of introduced eastern grey squirrels on red squirrel population dynamics. Journal of Animal Ecology 73: 26–35. Available: http://doi.wiley.com/10.1111/j.1365-2656.2004.00791.x. Accessed 7 July 2012.

[38] Lawton C, Cowan P, Bertolino S, Lurz PWW, Peters AR (2010) The consequences of introducing non-indigenous species: two case studies, the grey squirrel in Europe and the brushtail possum in New Zealand. Revue Scientifique et Technique - Office International des Épizooties 29: 113–122. 10.20506/rst.29.2.1983 20919583

[39] Dagnall J, Gurnell J, Pepper H (1998) Bark-stripping by gray squirrels in state forests of the United Kingdom: a review. In: Steele MA, Merritt JF, Zegers DA, editors. Ecology and evolutionary biology of tree squirrels. Special Publication, Virginia Museum of Natural History, Martinsville, USA. 249–261.

[40] Newson S, Leech D, Hewson C, Crick H, Grice P (2010) Potential impact of grey squirrels Sciurus carolinensis on woodland bird populations in England. Journal of Ornithology 151: 211–218. Available: http://dx.doi.org/10.1007/s10336-009-0445-8. Accessed 18 May 2013.

[41] Rushton SP, Lurz PWW, Fuller R, Garson PJ (1997) Modelling the distribution of the red and grey squirrel at the landscape scale: a combined GIS and population dynamics approach. Journal of Applied Ecology 34: 1137–1154. Available: http://www.jstor.org/stable/2405227. Accessed 18 May 2013.

[42] Lurz PWW, Rushton SP, Wauters LA, Bertolino S, Currado I, et al. (2001) Predicting grey squirrel expansion in North Italy: a spatially explicit modelling approach. Landscape Ecology 16: 407–420. Available: http://dx.doi.org/10.1023/A:1017508711713. Accessed 18 May 2013.

[43] Tattoni C, Preatoni D, Lurz PWW, Rushton SP, Tosi G, et al. (2006) Modelling the Expansion of a Grey Squirrel population: Implications for Squirrel Control. Biological Invasions 8: 1605–1619. Available: http://dx.doi.org/10.1007/s10530-005-3503-z. Accessed 18 May 2013.

[44] Bertolino S, Lurz PWW, Sanderson R, Rushton SP (2008) Predicting the spread of the American grey squirrel (Sciurus carolinensis) in Europe: A call for a co-ordinated European approach. Biological Conservation 141: 2564–2575. Available: http://www.sciencedirect.com/science/article/pii/S0006320708002723. Accessed 18 May 2013.

[45] Guralnick RP, Wieczorek J, Beaman R, Hijmans RJ (2006) BioGeomancer: Automated Georeferencing to Map the World’s Biodiversity Data. PLoS Biology 4: 1908–1909. Available: http://dx.doi.org/10.1371/journal.pbio.0040381. Accessed 18 May 2013. 10.1371/journal.pbio.0040381 PMC1637066 17105347

[46] IUCN (2012) IUCN Red List of Threatened Species. Version 2012.1.

[47] Thuiller W (2004) Patterns and uncertainties of species’ range shifts under climate change. Global Change Biology: 2020–2027. Available: http://onlinelibrary.wiley.com/doi/10.1111/j.1365-2486.2004.00859.x/full. Accessed 6 May 2013.

[48] Franklin J (1995) Predictive vegetation mapping: geographic modelling of biospatial patterns in relation to environmental gradients. Progress in Physical Geography. Available: http://ppg.sagepub.com/content/19/4/474.short. Accessed 6 May 2013.

[49] McGill B (2010) Matters of scale. Science 328: 575–576. Available: http://www.montana.edu/hansen/documents/labreadings2011/McGill 2010.pdf. Accessed 6 May 2013. 10.1126/science.1188528 20431001

[50] Hijmans RJ, Cameron SE, Parra JL, Jones PG, Jarvis A (2005) Very high resolution interpolated climate surfaces for global land areas. International Journal of Climatology 25: 1965–1978. Available: http://dx.doi.org/10.1002/joc.1276. Accessed 18 May 2013.

[51] Gurnell J (1996) The effects of food availability and winter weather on the dynamics of a grey squirrel population in southern England. Journal of Applied Ecology 33: 325–328.

[52] Nixon CM, McClain MW (1969) Squirrel population decline following a late spring frost. Journal of Wildlife Management 33: 353–357.

[53] Middleton AD (1932) The Grey Squirrel (Sciurus carolinensis) in the British Isles, 1930–1932. Journal of Animal Ecology 1: 166–167.

[54] Guisan A, Thuiller W (2005) Predicting species distribution: offering more than simple habitat models. Ecology Letters 8: 993–1009. Available: http://doi.wiley.com/10.1111/j.1461-0248.2005.00792.x. Accessed 29 February 2012. 10.1111/j.1461-0248.2005.00792.x 34517687

[55] R Development Core Team (2012) R: A language and environment for statistical computing. Vienna, Austria: R Foundation for Statistical Computing.

[56] Phillips SJ, Anderson RP, Schapire RE (2006) Maximum entropy modeling of species geographic distributions. Ecological Modelling 190: 231–259. Available: http://linkinghub.elsevier.com/retrieve/pii/S030438000500267X. Accessed 8 March 2012.

[57] Phillips SJ, Dudík M (2008) Modeling of species distributions with Maxent: new extensions and a comprehensive evaluation. Ecography 31: 2005–2010. Available: http://onlinelibrary.wiley.com/doi/10.1111/j.0906-7590.2008.5203.x/full. Accessed 19 June 2012.

[58] Jaynes ET (1957) Information Theory and Statistical Mechanics. II. Physical Review 108: 171–190. Available: http://link.aps.org/doi/10.1103/PhysRev.108.171. Accessed 18 May 2013.

[59] Elith J, Graham C, Anderson R, Dudík M (2006) Novel methods improve prediction of species’ distributions from occurrence data. Ecography 29: 129–151. Available: http://onlinelibrary.wiley.com/doi/10.1111/j.2006.0906-7590.04596.x/full. Accessed 19 June 2012.

[60] Heikkinen RK, Luoto M, Araújo MB, Virkkala R, Thuiller W, et al. (2006) Methods and uncertainties in bioclimatic envelope modelling under climate change. Progress in Physical Geography 30: 751–777. Available: http://ppg.sagepub.com/content/30/6/751.abstract. Accessed 18 May 2013.

[61] Ficetola GF, Thuiller W, Miaud C (2007) Prediction and validation of the potential global distribution of a problematic alien invasive species – the American bullfrog. Diversity and Distributions 13: 476–485. Available: http://dx.doi.org/10.1111/j.1472-4642.2007.00377.x. Accessed 18 May 2013.

[62] Freeman E (2007) PresenceAbsence: An R Package for Presence-Absence Model Evaluation. USDA Forest Service, Rocky Mountain Research Station. 507 25th street, Ogden, UT, USA.

[63] Broennimann O, Fitzpatrick MC, Pearman PB, Petitpierre B, Pellissier L, et al. (2011) Measuring ecological niche overlap from occurrence and spatial environmental data. Global Ecology and Biogeography 21: 481–497. Available: http://doi.wiley.com/10.1111/j.1466-8238.2011.00698.x. Accessed 9 March 2012.

[64] Silverman BW (1986) Density estimation for statistics and data analysis. London, United Kingdom: Chapman and Hall.

[65] Schoener TW (1970) Nonsynchronous spatial overlap of lizards in patchy habitats. Ecology 51: 408–418.

[66] Warren DL, Glor RE, Turelli M (2008) Environmental niche equivalency versus conservatism: quantitative approaches to niche evolution. Evolution 62: 2868–2883 doi:10.1111/j.1558-5646.2008.00482.x 18752605 10.1111/j.1558-5646.2008.00482.x

[67] Swets JA (1988) Measuring the accuracy of diagnostic systems. Science 240: 1285–1293. Available: http://www.sciencemag.org/content/240/4857/1285.abstract. Accessed 18 May 2013. 10.1126/science.3287615 3287615

[68] Allouche O, Tsoar A, Kadmon R (2006) Assessing the accuracy of species distribution models: prevalence, kappa and the true skill statistic (TSS). Journal of Applied Ecology 43: 1223–1232. Available: http://dx.doi.org/10.1111/j.1365-2664.2006.01214.x. Accessed 18 May 2013.

[69] Thuiller W, Lavorel S, Araújo MB (2005) Niche properties and geographical extent as predictors of species sensitivity to climate change. Global Ecology and Biogeography 14: 347–357. Available: http://dx.doi.org/10.1111/j.1466-822X.2005.00162.x. Accessed 18 May 2013.

[70] Haagner A (1920) South African mammals. Witherby HF, Witherby G, editors London, United Kingdom.

[71] Lever C (1985) Naturalized mammals of the World. Longman, editor London, United Kingdom.

[72] Broennimann O, Guisan A (2008) Predicting current and future biological invasions: both native and invaded ranges matter. Biology Letters 4: 585–589. Available: http://www.pubmedcentral.nih.gov/articlerender.fcgi?artid=2610080&tool=pmcentrez&rendertype=abstract. Accessed 1 March 2012. 10.1098/rsbl.2008.0254 PMC2610080 18664415

[73] Rödder D, Lötters S (2010) Explanative power of variables used in species distribution modelling: an issue of general model transferability or niche shift in the invasive Greenhouse frog (Eleutherodactylus planirostris). Naturwissenschaften 97: 781–796. Available: http://www.springerlink.com/index/L305340876177312.pdf. Accessed 20 June 2012. 10.1007/s00114-010-0694-7 20617298

[74] Botkin DB, Saxe H, Araújo MB, Betts R (2007) Forecasting the effects of global warming on biodiversity. Bioscience 57: 227–236. Available: http://www.bioone.org/doi/abs/10.1641/B570306. Accessed 7 July 2012.

[75] Hof C, Levinsky I, Araújo MB, Rahbek C (2011) Rethinking species’ ability to cope with rapid climate change. Global Change Biology 17: 2987–2990. Available: http://doi.wiley.com/10.1111/j.1365-2486.2011.02418.x. Accessed 1 March 2012.

[76] Steele MA, Koprowski JL (2001) North American Tree Squirrels. Smithsonian Institution Press.

[77] Lloyd HG (1983) Past and present distribution of Red and Grey squirrels. Mammal Review 13: 69–80.

