# Supplementary material for: The Use of Climatic Niches in Screening Procedures for Introduced Species to Evaluate Risk of Spread: A Case with the American Eastern Grey Squirrel
Source: PLoS One. 2013 Jul 3;8(7):e66559. doi: 10.1371/journal.pone.0066559 (PMC3701016; doi:10.1371/journal.pone.0066559)
Supplement: Table S1 — Results of niche shift analysis performed using all ordination and SDMs methods proposed by Broennimann et al., (2011). “*” indicates methods calibrated in native range and projected in invasive range. D metric quantifies niche overlap (Schoener, 1970). Arrows specify if a niche similarity test was performed, simulating niches in range 1 and calculating niche overlap with observed niche in range 2, or vice versa. (DOCX) [file pone.0066559.s001.docx]

**SUPPORTING INFORMATION**

**Table S1**

| **Technique** | **Area of calibration** | ***D* metric** | **Niche equivalency test significance level** | **Niche similarity test (range 1🡪range 2) significance level** | **Niche similarity test (range 1🡨range 2) significance level** |
| --- | --- | --- | --- | --- | --- |
| PCA – occ | Occurrences in North America + UK, Ireland, Piedmont | 0.183 | < 0.01 | ns | ns |
| PCA – occ* | Occurrences in North America | 0.201 | < 0.01 | ns | ns |
| PCA – env | Native + Invasive range | 0.208 | < 0.01 | ns | ns |
| PCA – env* | Native range | 0.243 | < 0.01 | ns | ns |
| BETWEEN – occ | Occurrences in North America + UK, Ireland, Piedmont | 0.158 | < 0.01 | ns | ns |
| WITHIN – occ | Occurrences in North America + UK, Ireland, Piedmont | 0.311 | < 0.01 | < 0.05 (sim) | < 0.05 (sim) |
| WITHIN – env | Native + Invasive range | 0.072 | < 0.01 | ns | ns |
| LDA | Occurrences in North America + UK, Ireland, Piedmont | 0.408 | < 0.01 | ns | ns |
| MDS | Occurrences in North America + UK, Ireland, Piedmont | 0.174 | < 0.01 | ns | ns |
| MDS* | Occurrences in North America | 0.209 | < 0.01 | ns | ns |
| MAXENT | Occurrences in North America + UK, Ireland, Piedmont | 0.523 | < 0.01 | < 0.01 (sim) | < 0.01 (sim) |
